# A Comparative Analysis of the Immunoglobulin Repertoire in Leukemia Cells and B Cells in Chinese Acute Myeloid Leukemia by High-Throughput Sequencing

**DOI:** 10.3390/biology13080613

**Published:** 2024-08-13

**Authors:** Huige Yan, Lina Wu, Pingzhang Wang, Miaoran Xia, Zhan Shi, Xinmei Huang, Sha Yin, Qian Jiang, C. Cameron Yin, Xiangyu Zhao, Xiaoyan Qiu

**Affiliations:** 1 Department of Immunology, School of Basic Medical Sciences, Peking University, Beijing 100191, China; yhg@pku.edu.cn (H.Y.); wangpzh@bjmu.edu.cn (P.W.); shi-zhan@pku.edu.cn (Z.S.); huangxm2017@pku.edu.cn (X.H.); shayin@bjmu.edu.cn (S.Y.); 2 Key Laboratory of Immunology, National Health Commission, Beijing 100191, China; 3 Key Laboratory of Carcinogenesis and Translational Research (Ministry of Education/Beijing), Central Laboratory, Peking University Cancer Hospital & Institute, Beijing 100142, China; lnwu@bjmu.edu.cn; 4 Department of Immunology, Capital Medical University, Beijing 100069, China; mxia@ccmu.edu.cn; 5 Peking University Institute of Hematology, Beijing Key Laboratory of Hematopoietic Stem Cell Transplantation, National Clinical Research Center for Hematologic Disease, Peking University People’s Hospital, No. 11 South Street of Xizhimen, Xicheng District, Beijing 100044, China; jiangqian@medmail.com.cn; 6 Department of Hematopathology, The University of Texas MD Anderson Cancer Center, Houston, TX 77030, USA

**Keywords:** acute myeloid leukemia, myeloblast, immunoglobulin, V(D)J rearrangement patterns, somatic hypermutation, B cells

## Abstract

**Simple Summary:**

It is known that both B cells and myeloblasts from acute myeloid leukemia (AML) express immunoglobulin (Ig). However, the difference between Ig from myeloblasts and B cells has not been explored; therefore, the function and significance of AML-Ig are not well known. Here, we performed 5′ RACE-related PCR coupled with PacBio sequencing to analyze the Ig repertoire of Chinese AML patients. Myeloblasts expressed all five classes of IgH, especially Igγ, with a high expression frequency. Compared with B-Ig in the same AML patient, AML-Ig showed different biased V(D)J usages and mutation patterns. More importantly, mutations of AML-Ig frequently occurred at the sites of post-translational modification. In summary, our results showed that AML-Ig had different sequence characteristics than those of B-Ig and that the unique V(D)J usages of AML-Ig may serve as a novel biomarker for personalized minimal/measurable residual disease monitoring and therapeutic targets in AML patients.

**Abstract:**

It is common knowledge that immunoglobulin (Ig) is produced by B lymphocytes and mainly functions as an antibody. However, it has been shown recently that myeloblasts from acute myeloid leukemia (AML) could also express Ig and that AML-Ig played a role in leukemogenesis and AML progression. The difference between Ig from myeloblasts and B cells has not been explored. Studying the characteristics of the Ig repertoire in myeloblasts and B cells will be helpful to understand the function and significance of AML-Ig. We performed 5′ RACE-related PCR coupled with PacBio sequencing to analyze the Ig repertoire in myeloblasts and B cells from Chinese AML patients. Myeloblasts expressed all five classes of IgH, especially Igγ, with a high expression frequency. Compared with B-Ig in the same patient, AML-Ig showed different biased V(D)J usages and mutation patterns. In addition, the CDR3 length distribution of AML-Ig was significantly different from those of B-Ig. More importantly, mutations of AML-IgH, especially Igμ, Igα, and Igδ, were different from that of B-IgH in each AML patient, and the mutations frequently occurred at the sites of post-translational modification. AML-Ig has distinct characteristics of variable regions and mutations, which may have implications for disease monitoring and personalized therapy.

## 1. Introduction

It is well known that B lymphocytes are the origin of immunoglobulin (Ig), which mainly functions as an antibody. However, in recent years, growing evidence has revealed that non-B cells, including myeloid cells, can also produce Ig [1,2,3,4,5,6,7,8]. Non-B-derived Igs have been shown to be highly expressed in cancer cells, including breast [9], colon [3], lung [10,11], prostate [12], esophagus [13], pancreas [14], and bladder cancers, as well as some soft tissue tumors [15]. Functionally, these Igs play a role in tumorigensis by stimulating tumor cell growth, survival, and metastasis [1,10,11] or promoting immune escape [16].

In fact, as early as 1993, Schmetzer et al. found clonal Ig gene rearrangements in newly diagnosed AML patients by Southern blot [7,8]. However, it has remained unclear whether there are products of Ig genes in AML cells until 2013, when our group first reported that myeloid cells, including myeloblasts, expressed both the transcripts and proteins of IgG, IgM, and IgK [4,5,17]. Moreover, high levels of IgG expression were associated with monocytic differentiation, multilineage dysplasia, *TET2* and *KRAS* mutations, and poor overall survival [17]. Both IgG and IgM promote myeloblast proliferation [4]. These results suggested that AML-derived Ig may have a role in AML pathogenesis and progression and serve as a novel biomarker for disease monitoring and target therapy.

Sequence analysis of Ig genes has provided valuable information that is essential to the understanding of the prognosis of B cell lymphomas [18]. Mutation status of the Ig heavy chain variable region genes (IGHV) has a major impact on the prognosis of chronic lymphocytic leukemia (CLL)/small lymphocytic lymphoma and has implications for treatment stratification in clinical trials [18,19,20,21]. Hairy cell leukemia (HCL), a rare type of indolent B cell leukemia different from CLL, and Ig somatic hypermutation (SHM) in the IGHV gene are present in 80–90% of patients with HCL [22]. Features of canonical SHM include (1) an increased ratio of mutations that change (replace) versus those that do not change (silent) the amino acid sequence (replacement-to-silent [R/S]), particularly in the complementarity determining regions (CDRs) as opposed to framework regions (FRs), (2) an increased ratio of transitions (purine to purine or pyrimidine to pyrimidine) to transversions, and (3) more than the expected percentage of mutations in the RGYW hot spots relative to the total number of nucleotides located within these hot spots [23]. Unmutated IGHV genes have been associated with resistance to single-agent cladribine in patients with HCL and have been used as a tool to identify a minor group of refractory patients [24]. Therefore, we hypothesize that AML-derived-Ig (AML-Ig) may have unique structural and functional characteristics that are different from B cell Ig, which may provide a basis for using AML-Ig for monitoring of minimal/measurable residual disease (MRD) and the development of target therapy.

A few earlier studies explored the characteristics of Ig rearrangements in AML. Most of these studies used Sanger sequencing to assess AML-Ig rearrangements and found clues for the biased expression and conservation [4,5,25]. Subsequently, by multiplex PCR combined with next generation sequencing (NGS), we discovered that VDJ rearrangements of Ig heavy chains in myeloblasts were biased with individual specificity rather than diverse as seeb in B cells [26]. Furthermore, AML-derived IGK was more conserved among different AML samples, and it differed from classical IGK in B cells in the rate of hypermutation and special mutation hotspots at the serine site [26]. However, some limitations were found in the previous studies. Firstly, the B cell controls were from a pool of healthy individuals instead of the same individual. Secondly, there was a possibility of biased amplification due to multiplex PCR.

In this study, we used a 5′ RACE-related PCR coupled with the latest PacBio sequencing (a technique of third-generation sequencing) to analyze the heavy-chain repertoire of Ig in leukemia cells and B cells from newly diagnosed Chinese AML patients with no prior treatment. The results showed that the biased V(D)J usage patterns of AML-Ig were different from those of B-Ig from the same patient. More importantly, mutations of AML-IgH, especially Igμ, Igα, and Igδ, frequently occurred at the sites of post-translational modification, different from B-IgH, in the same individual AML patients. The biased V(D)J usage and the unique mutation patterns of AML-Ig may serve as targets for detecting MRD in AML.

## 2. Materials and Methods

### 2.1. AML Patient Samples

Bone marrow samples from AML patients (n = 27) were provided by Peking University People’s Hospital from May 2017 to May 2018. All AML patients were newly diagnosed and had not received any treatment before entry into the study.

### 2.2. Isolation and Preparation of Bone Marrow Mononuclear Cells

Mononuclear cells (MNCs) were prepared by Percoll density centrifugation. The white gradient layer containing bone marrow mononuclear cells (BMMCs) was isolated and washed with phosphate buffer solution (PBS). The isolated BMMCs were frozen in liquid nitrogen before cell sorting. This study was approved by the Institutional Review Board of Peking University (protocol code: IRB00001052-21005).

### 2.3. Fluorescence-Activated Cell Sorting

To sort the myeloblasts from AML patients, BMMCs were resuscitated, washed in PBS, and blocked with 5% fetal bovine serum (FBS) for 30 min on ice. The BMMCs were then divided into two groups. One group was stained with mouse anti-human CD33 PerCP (eBioscience/Thermo Fisher, Waltham, MA, USA), mouse anti-human CD138 PE (BD Pharmingen, Franklin Lakes, NJ, USA), and mouse anti-human CD19 APC (Miltenyi Biotec, Bergisch Gladback, Garmany) for 30 min on ice in the dark. The other group was stained with mouse anti-human CD45 APC-Vio770 (Miltenyi Biotec), mouse anti-human CD34 VioBlue (Miltenyi Biotec), mouse anti-human CD19 APC (Miltenyi Biotec), and mouse anti-human CD14 Percp (Miltenyi Biotec) for 30 min on ice in the dark. Next, we performed flow cytometry cell sorting for the above two groups of cells to achieve different experimental aims. For the former group, we collected CD45dim+CD33+CD19-CD138- cells (myeloblasts) and CD45+CD33-CD19+ (B cells) to compare the Ig repertoire between myeloblasts and B cells. For the latter group, we collected CD45dim+CD34+CD14-CD19- myeloid progenitor cells (myeloblasts) and CD45+CD34-CD14+CD19- mature monocytes to compare the Ig repertoire in myeloid cells at different stages of differentiation. CD45+CD19+CD34- B cells were used as a control.

### 2.4. Flow Cytometry Analysis

In order to analyze the expression of Igs on the surface of myeloblasts, BMMCs were stained with mouse anti-human IgG BV605 (BD Pharmingen), mouse anti-human IgM APC/Cy7 (BD Pharmingen), mouse anti-human Igκ VioBright-FITC (Miltenyi Biotec), and mouse anti-human Igλ APC (BD Pharmingen) and analyzed using a FACScan cytometer.

### 2.5. RNA Extraction and cDNA Synthesis by 5′-RACE

Total RNA was extracted from sorted myeloblasts using the RaPure Total RNA Micro Kit (Magen, Guangzhou, China) according to the manufacturer’s instructions. 5′-RACE cDNA synthesis was performed using the SMARTer^®^ RACE 5′/3′ Kit (Takara, San Jose, CA, USA) and a complete cDNA copy with an additional specific sequence at the 5′ end was generated.

### 2.6. Nested PCR

The variable regions of Igs were amplified by a nested PCR. To amplify complete Ig V(D)J regions from 5′-RACE cDNA, we used the upstream primer targeted additional specific sequence from 5′-RACE and the downstream primer targeted constant-region for Igγ, α, ε, μ, and δ in both the first-round and second-round PCR. In particular, barcodes were added to the second-round PCR primers for PacBio sequencing to split the sequencing results. The PCR program for both rounds was 35 cycles (first-round PCR) or 40 cycles (second-round PCR) at 94 °C for 30 s, 68 °C for 30 s, and 72 °C for 2 min. In addition, we performed PCR to assess B cell genes (CD19, CD20) and GAPDH using the following conditions: 95 °C for 5 min, 40 cycles at 95 °C for 30 s, 56 °C for 30 s, and 72 °C for 30 s, followed by a final extension at 72 °C for 10 min. The PCR products were separated on a 1.5% agarose gel by electrophoresis. The sequences of the primers used for all PCR reactions are listed in Supplemental Table S1.

### 2.7. PacBio Sequencing and Data Analysis

The PCR products with barcodes were quantified and mixed with the same amount of PCR products of Ig from myeloblasts, monocytes, or B cells. The mixed PCR products were separated on 2% agarose gel by electrophoresis, extracted by the TIANgel Purification Kit (TIANGEN, Beijing, China), and 3 μg products were used for PacBio sequencing. The data was obtained based on barcodes using the FASTX-toolkit (version 0.0.13). IMGT/High V-QUEST (version 1.7.1) was used for sequence annotation to determine the V(D)J genes, CDRs, junctional modifications, and characteristic mutations in the variable regions.

### 2.8. Protein Extraction and Western Blot Analysis

The sorted CD33^+^ and CD34^+^ myeloblasts were washed twice with ice-cold PBS and lysed in RIPA lysis buffer (Leagene, Beijing, China) with freshly prepared proteinase inhibitor cocktail (Roche, Basel, Switzerland) for 30 min at 4 °C. Cell lysate was clarified by centrifugation at 4 °C at 16,000× *g* for 10 min. Protein concentration was determined using the BCA protein assay reagent (Invitrogen, Waltham, MA, USA). Equal amounts of proteins were separated by 12% SDS-PAGE and transferred onto nitrocellulose membranes (Whatman, Little Marlow, UK). Membranes were blocked in Tris-buffered saline containing 0.1% Tween-20 (TBST) and 5% nonfat milk for 1 h and incubated with the appropriate primary antibody overnight at 4 °C. After washing in TBST buffer, membranes were incubated for 1 h with the appropriate secondary antibodies. Signals were detected on the ImageQuant LAS 500 imager (GE Healthcare Life Sciences, Marlborough, MA, USA).

### 2.9. Statistical Analysis

All data were analyzed with GraphPad Prism software version 5 and presented as mean ± SD. The correlation between Ig expression and mutation in different cell types was tested by the chi-square test. The comparison of Ig expressions was performed by the Wilcoxon matched-pairs signed rank test and the Mann-Whitney test. Statistical significance was defined as * *p* < 0.05, ** *p* < 0.01, *** *p* < 0.001, or **** *p* < 0.0001.

## 3. Results

### 3.1. Characteristics of AML Patients and the Strategy of Ig Sequencing

Bone marrow samples from AML patients (n = 27) were obtained from Peking University People’s Hospital. All patients were newly diagnosed and had not received any treatment. The diagnosis was made based on the 2022 World Health Organization classification of hematopoietic neoplasms. The percent of myeloblasts ranged from 25% to 86%. The detailed clinicopathologic, cytogenetic, and molecular features of 27 AML patients (AML1-27) are listed in Table 1.

**Figure 1:**
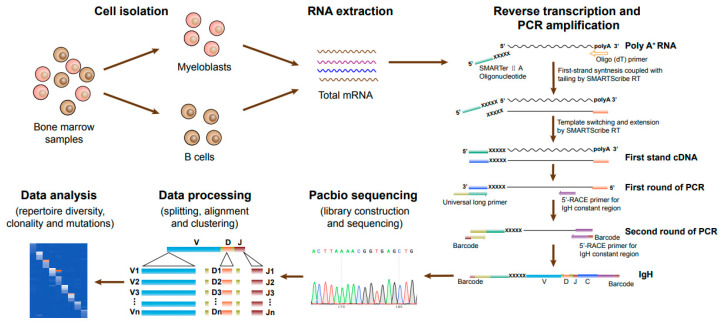
Schematic of steps involved in PacBio sequencing of Ig genes from myeloblasts and B cells. Bone marrow mononuclear cells were obtained from AML patients. Myeloblasts and B cells were sorted by fluorescence-activated cell sorting. Total mRNA was extracted and reverse transcribed by 5′-RACE, and the Ig heavy chain and light chain were amplified by multiplex PCR. Through library construction, PacBio sequencing, and data processing, the Ig repertoire was obtained and analyzed.

To compare Igs from myeloblasts and B cells, we sorted myeloblasts and B cells from the bone marrow of 17 AML patients (AML1-17) using flow cytometry. The 5′-RACE provides a mechanism for adding special nucleotide sequences to the 5′ end of the cDNA [27]. The schematic of steps involved in PacBio sequencing of Ig genes from myeloblasts and B cells is shown in Figure 1. The upstream and downstream PCR primers were designed to target the 5′ end specific nucleotide sequences and Ig constant regions to generate full-length V(D)J sequences. The transcripts of all five classes of IgH were obtained through 5′-RACE and nested PCR, and the Ig repertoire that contained 5′ non-translated regions and V-(D)-J-C sequences were obtained by PacBio sequencing, which were analyzed by IMGT/HighV-QUEST (version 1.7.1) [28].

### 3.2. Primary Myeloblasts Express Different Types of Ig Heavy Chains

**Figure 2:**
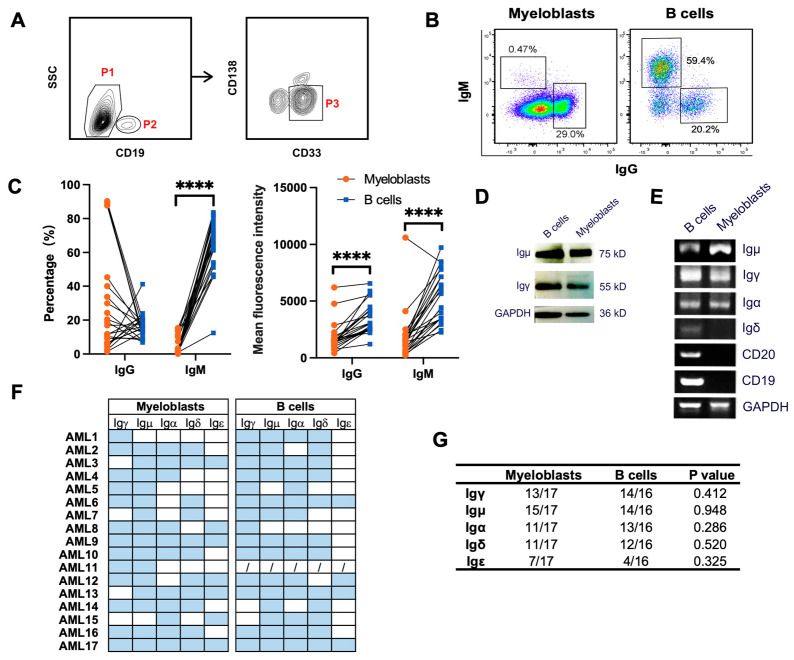
Different classes and types of Ig expressed in primary myeloblasts. (**A**) Representative gating strategy for myeloblasts. Bone marrow mononuclear cells were obtained from AML patients. Myeloblasts (P1 and P3 gates, CD45^+^CD19^−^CD138^−^CD33^+^) and B cells (P2 gate, CD45^+^CD19^+^) were sorted by FACS. (**B**) Flow cytometry plots showing the gating strategy to detect IgG and IgM on the surface of myeloblasts and B cells. (**C**) Quantification of IgG and IgM in myelolasts and B cells from AML patients. Comparisons between myeloblasts and B cells were performed by the Wilcoxon matched-pairs signed rank test. **** *p* < 0.0001. (**D,E**) Western blot (**D**) and PCR (**E**) detection of Ig in myeloblasts and B cells. (**F**) Statistical analysis of the expression of Ig in myeloblasts and B cells from 17 AML patients. The blue box represents Ig expression, the white box represents no Ig expression, and the forward slash indicates that the samples were not assessed. (**G**) Quantification of Ig expression frequency in myeloblasts and B cells, and the association between cell types and Ig expression were assessed using the chi-square test.

We have shown previously that primary myeloblasts could express the transcripts of IgG, IgM, and Igκ [4,5,25]. Here, we detected protein expression of IgG and IgM on the cell membrane of CD33^+^ myeloblasts (CD19^−^CD33^+^ CD138^−^) from AML patients by flow cytometry, with B cells (CD19^+^SSC^low^) as the control (Figure 2A). Both IgG and IgM were expressed on the cell membrane of myeloblasts (Figure 2B). Whereas the frequency of IgM expressed on the surface of myeloblasts (mean ± SD, 4.2% ± 4.9%) was much lower than that on the surface of B cells (mean ± SD, 63.3% ± 15.9%), the frequency of IgG expressed on the surface of myeloblasts (mean ± SD, 28.6% ± 30.1%) was not significantly different from that on the B cells (mean ± SD, 16.2% ± 7.1%) (Figure 2C, left panel). The mean fluorescence intensity of both IgG and IgM on the surface of myeloblasts was lower than that on B cells (Figure 2C, right panel).

To further verify Ig expression profiles in myeloblasts, CD33^+^ primary myeloblasts and B cells from 17 AML patients were sorted by flow cytometry (Figure 2A). The expression of Igγ at both the protein level and transcription level was detected in myeloblasts and B cells by Western Blot (Figure 2D) and RT-PCR (Figure 2E), respectively. In addition, we analyzed the expression frequency of AML-Ig in 17 AML patients and detected high expression of Igμ (15/17, 88.2%), Igγ (13/17, 76.5%), Igα (11/17, 64.7%), and Igδ (11/17, 64.7%) in myeloblasts. As a comparison, the expression frequencies in B cells were: Igμ (14/16, 87.5%), Igγ (14/16, 87.5%), Igα (13/16, 81.3%), Igδ (12/16, 75.0%), and Igε (4/16, 25.0%). There was no significant difference between the expression frequency of different classes of IgH in myeloblasts and B cells (Figure 2F,G).

### 3.3. The Biased Usages of V(D)J Rearrangement Patterns in Myeloblasts Are Different from That of B Cells from the Same Patient

It is known that V(D)J rearrangements mainly form the Ig V region sequences and can specifically bind to unique antigens. Through PacBio sequencing, IgH sequences from the 5’-RACE PCR product were obtained from myeloblasts and B cells, including Igγ (n = 78,672), Igμ (n = 31,354), Igα (n = 50,651), Igδ (n = 34,400), and Igε (n = 9099) (Table 2). The Igε sequences were only detected in one case of myeloblasts and four cases of B cells; their sequence characteristics were therefore not analyzed.

**Figure 3:**
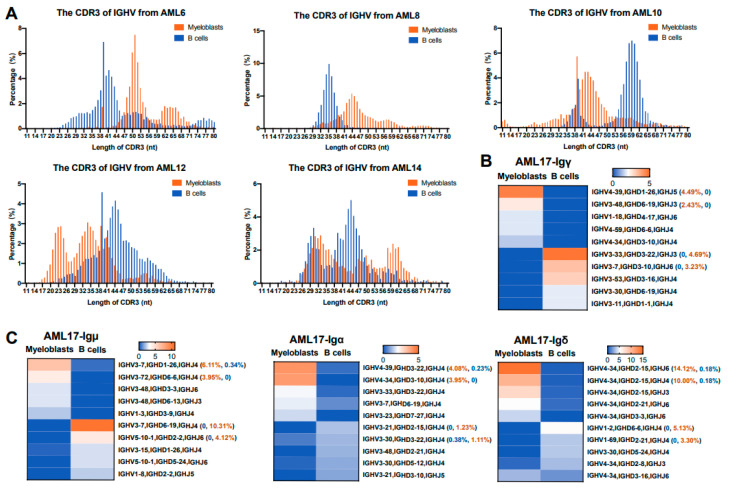
The CDR3 distribution and the restricted V(D)J rearrangement patterns of Ig in myeloblasts and B cells. (**A**) Length distribution of CDR3 sequences in IgH from five AML patients. The *x*-axis shows the length of CDR3. (**B,C**) The top five V(D)J rearrangement patterns used by myeloblasts and B cells. Heat maps showing the expression frequency of four classes of Ig heavy chains, including γ (**B**), μ, α, and δ chains (**C**).

The complementarity-determining region 3 (CDR3), where the distal portion of the V segment joins the (D)J segment, is the center of the antigen-binding site. In B cells, the high diversity of the length and sequence of the CDR3 region leads to a huge diversity of Ig [29]. We compared the CDR3 characteristics of AML-Ig with those of B-Ig in the same AML patient and found that the CDR3 length distribution of IgH (Figure 3A and Supplementary Figure S1) in myeloblasts was obviously different from that of B cells. These results suggest that the CDR3 length distribution of AML-IgH is different from that of B cells.

We also compared the characteristics of V_H_DJ_H_ rearrangement patterns in myeloblasts and B cells from the same AML patient and found that V_H_DJ_H_ rearrangements in myeloblasts showed biased usage, and the biased V_H_DJ_H_ rearrangement patterns were different from those of B cells in the same patient. We further confirmed these results in all classes of IgH, including Igγ, Igμ, Igɑ, and Igδ, from multiple AML patients (Figure 3B,C, Supplementary Figures S2 and S3). Take AML 17 as an example, IGHV4-39/IGHD1-26/IGHJ5 in Igγ, IGHV3-7/IGHD1-26/IGHJ4 in Igμ, IGHV4-39/IGHD3-22/IGHJ4 in Igα, and IGHV4-34/IGHD2-15/IGHJ6 in Igδ were among the highest in usages in myeloblasts. However, the highest usages of VDJ rearrangement patterns of B-Ig from the same patient were IGHV3-33/IGHD3-22/IGHJ3 in Igγ, IGHV3-7/IGHD6-19/IGHJ4 in Igμ, IGHV3-21/IGHD2-15/IGHJ4 in Igα, and IGHV1-2/IGHD6-6/IGHJ4 in Igδ. These results indicated that there were no class switch phenomena in AML-Ig, and the data also confirmed that sorted myeloblasts were free of B cell contamination because the V_H_DJ_H_ rearrangement patterns between B cells and myeloblasts were different. Furthermore, we assessed the distribution of V_H_ genes in the genomes of AML-Ig and B-Ig and found that V_H_ genes from AML-Ig were different from those of B-Ig. In general, the V_H_ genes that were closer to the J_H_ region in the genome were used more frequently in myeloblasts than in B cells, likely due to the fact that the closer spatial distance may be easier to selecte during Ig rearrangement in myeloblasts. (Supplementary Figures S4–S6). The data indicated that myeloblasts tended to employ the V segment closer to the J region in the genome, suggesting that the open chromatin state of AML-Ig may be distinct from that of B-Ig.

### 3.4. IGHV Mutations with Relatively High Frequency Occur at Unique Sites in Myeloblasts

**Figure 4:**
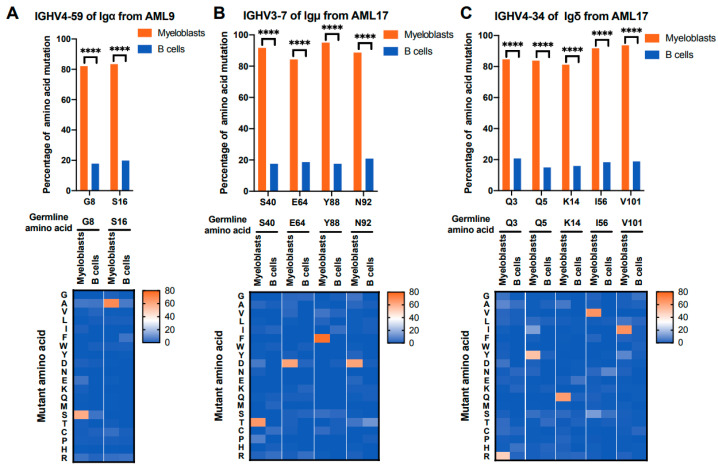
The highly frequent mutation sites in several special amino acids in V_H_ from myeloblasts. (**A**) Highly frequent mutation sites of IGHV4-59 of AML-Igα (n = 106) compared with B-Igα (n = 315) from AML9. (**B**) Highly frequent mutation sites of IGHV3-7 of AML-Igμ (n = 202) compared with B-Igμ (n = 90) from AML17. (**C**) Highly frequent mutation sites of IGHV4-34 of AML-Igδ (n = 2903) compared with B-Igδ (n = 196) from AML17. The *x*-axis shows the germline amino acid of V_H_ sequences, and the *y*-axis shows the percentage of mutation in AML-Ig and B-Ig (upper panel). Heat maps show the mutation states of amino acids. The *x*-axis shows the germline amino acids of V_H_ sequences, and the *y*-axis shows the mutant amino acids in AML-Ig and B-Ig (lower panel). The association between cell types and mutations was assessed by a chi-square test. **** *p* < 0.0001.

It is known that over 90% of AML patients harbor somatic gene mutations, which play a role in AML pathogenesis and progression [30,31,32,33]. To explore whether AML-Ig gene has unique mutations in the V region_,_ the mutation status of the V_H_ genes of both myeloblasts and B cells in 17 AML patients was evaluated. Our results showed that somatic mutations were detected in the AML-IgH, including Igγ, Igμ, Igα, and Igδ, and the mutations tended to occur at specific amino acid sites. For example, G8 and S16 in IGHV4-59 of AML-Igα showed a higher frequency of mutations (>80%) compared with that of B-Igα (<20%) in AML9 (Figure 4A, upper panel). Interestingly, the mutation sites with high frequencies in V_H_ tended to mutate into specific amino acids. G8 in IGHV4-59 of AML-Igα tended to mutate into S (63/106, 59.4%, 63/106) compared with that of B-Igα (15/315, 4.8%), while S16 in IGHV4-59 of AML-Igα tended to mutate into A (74/106, 69.8%) compared with that of B-Igα (20/315, 6.4%) in AML9 (Figure 4A, lower panel). Meanwhile, in AML17, the mutation frequencies of S50, E64, N92, and Y88 in IGHV3-7 of AML-Igμ were all higher than 80%, which was significantly higher than that of B cells (less than 20%), and these mutation sites tended to mutate into T (135/202, 66.8%), D (129/202, 63.9%), F (157/202, 77.7%), and D (129/202, 63.9%), respectively, compared with that of B-Igμ (less than 3%, n = 90) (Figure 4B). In addition, the same phenomenon was found in IGHV4-34 of AML-Igδ from AML17 (Figure 4C). These results indicated that high-frequency mutations of V_H_ occurred in some unique sites in each patient, which might affect the development of AML and serve as a marker for detecting MRD or a potential therapeutic target.

**Figure 5:**
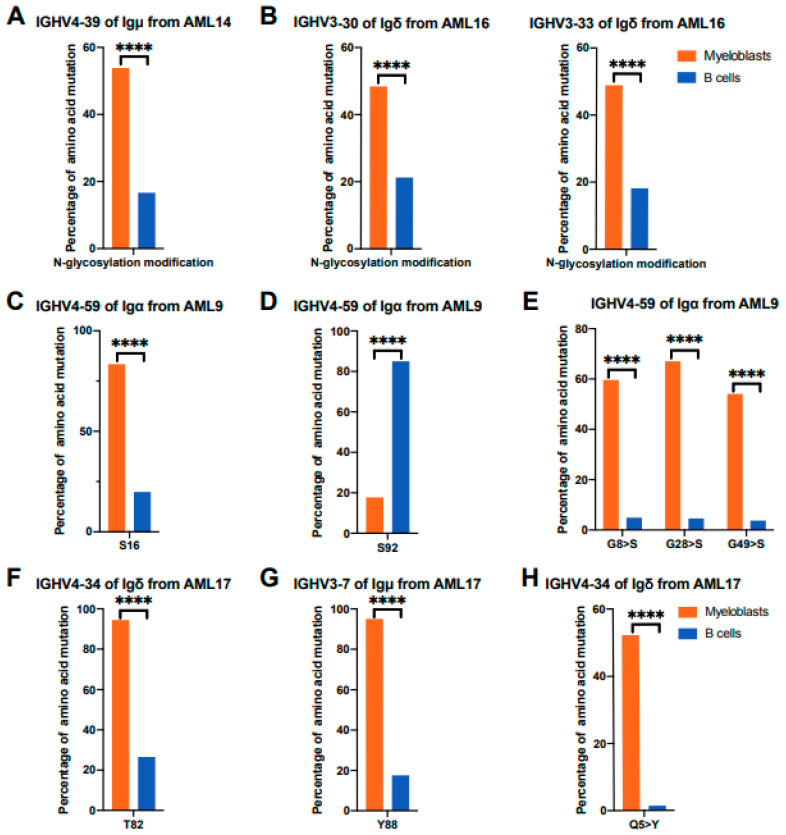
Comparisons of V_H_ mutation sites related to post-translational modifications between myeloblasts and B cells. (**A**) Proportion of V_H_ with potential N-glycosylation modification sites in myeloblasts and B cells. Comparison of IGHV4-39 of AML-Igμ (n = 39) and B-Igμ (n = 64) from AML14. (**B**) Comparison of IGHV3-30 of AML-Igδ (n = 832) and B-Igδ (n = 240) from AML16 (left panel). Comparison of IGHV3-33 of AML-Igδ (n = 1343) and B-Igδ (n = 121) from AML16 (right panel). (**C**–**E**) The mutation frequencies of S16 (**C**), S92 (**D**), and G8>S, G28>S, and G49>S (**E**) in IGHV4-59 of AML-Igα (n = 106) and B-Igα (n = 315) from AML9. S, serine; G, glycine. (**F**) The mutation frequency of T82 in IGHV4-34 of AML-Igδ (n = 2903) and B-Igδ (n = 196) from AML17. T, threonine. (**G**) The mutation frequency of Y88 in IGHV3-7 of AML-Igμ (n = 202) and B-Igμ (n = 90) from AML17. Y, tyrosine. (**H**) The mutation frequency of Q5>Y in IGHV4-34 of AML-Igδ (n = 2903) and B-Igδ (n = 196) from AML17. Q, glutamine. The association between cell types and mutations was tested by the chi-square test. **** *p* < 0.0001.

Immunoglobulin variable region gene mutations can undergo post-translational changes, which might directly influence antigen binding [34]. The post-translational modifications of Ig mainly include N-glycosylation [35,36,37], O-glycosylation, and tyrosine sulfation [38,39]. The potential N-glycosylation sites, which have the consensus sequence Asn-Xaa-Ser/Thr (where Xaa is not Pro), were analyzed. For example, IGHV4-39 of AML-Igμ had a higher proportion of potential N-glycosylation sites (21/39, 53.8%) than that of B-Igμ (11/64, 17.2%) in AML14 (Figure 5A). In addition, the proportion of potential N-glycosylation sites in IGHV3-30 (399/832, 48.0%) and IGHV3-33 (407/832, 48.9%) of AML-Igδ were higher than that in IGHV3-30 (51/240, 21.3%) and IGHV3-33 (43/240, 21.1%) of B-Igδ in AML16 (Figure 5B).

O-glycosylation modifications mainly occur on serine (S) or threonine (T). The amino acid mutation frequencies related to S or T of AML-IgH were different from B-IgH in AML patients. For example, S16 in IGHV4-59 of AML-Igα (88/106, 83.0%) had a higher mutation frequency than that of B-Igα (63/315, 20.0%) (Figure 5C), but S92 in IGHV4-59 of AML-Igα (19/106, 17.9%) had a lower mutation frequency than that of B-Igα (268/315, 85.1%) in AML9 (Figure 5D). Meanwhile, some amino acid sites in the V_H_ region were mutated into S, for example, the frequencies of G8 (63/106, 59.4%), G28 (71/106, 67.0%), and G49 (57/106, 53.8%) in IGHV4-59 of AML-Igα mutated to S were higher than G8 (15/315, 4.8%), G28 (14/315, 4.4%), and G49 (12/315, 3.8%) of B-Igα in AML9 (Figure 5E). Moreover, T82 in IGHV4-34 of AML-Igδ (2743/2903, 94.5%) had a higher mutation frequency than that of B-Igδ (52/196, 26.5%) in AML17 (Figure 5F). The mutations in S and T might affect potential O-glycosylation modifications in AML-IgH.

Tyrosine sulfation modifications mainly occur on tyrosine (Y) residues. The results showed that Y88 in IGHV3-7 of AML-Igμ (192/202, 95.1%) had a higher mutation frequency compared with B-Igμ (16/90, 17.8%) (Figure 5G). Another example was that the frequency of Q5 in IGHV4-34 of AML-Igδ (1515/2903, 52.2%) mutated to Y was higher than that of B-Igδ (3/196, 1.5%) in AML17 (Figure 5H). In addition, we also compared amino acid mutation frequencies and patterns on IGHVs between B-IGH from 11 healthy Chinese individuals (our unpublished database) and AML-IGH, and we found that these typical amino acid mutation frequencies and patterns in AML-IGH were also different from B-IGH of healthy donors (Supplementary Figure S7). These data suggested that Ig post-translational modifications, including N-glycosylation, O-glycosylation, and tyrosine sulfation, were different between myeloblasts and B cells.

### 3.5. Various Ig Classes of AML-Ig from the Same Patient Tend to Use Different V_H_DJ_H_ Rearrangement Patterns

**Figure 6:**
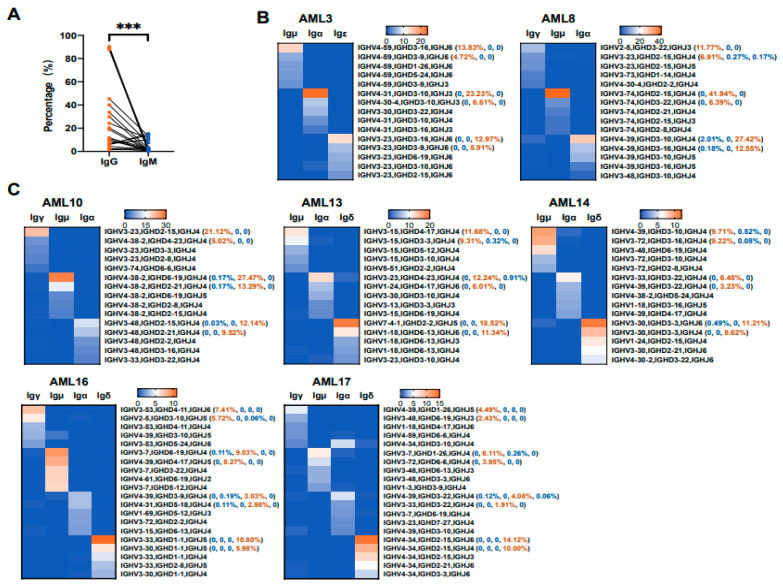
Characteristic Ig rearrangement patterns in different Ig classes expressed by myeloblasts from the same patient. (**A**) Flow cytometric quantification of IgG and IgM in myeloblasts from 22 AML patients. Comparisons between myeloblasts and B cells were calculated by the Wilcoxon matched-pairs signed rank test. *** *p* < 0.001. (**B**,**C**) Heat maps showing the top five V(D)J rearrangement patterns of four classes of heavy chains, including μ, γ, α, and δ chains, from the same individual from seven AML patients.

Upon encountering antigens, the naive B cells undergo class-switch recombination, in which exons encoding the default Cμ constant region of the IgH chain are excised and replaced with other constant region gene segments [40,41,42], leading to a switch in Ig expression from IgM to IgG, IgA, or IgE, whereas the rearranged V(D)J patterns remain unchanged [43,44]. It is known that the expression level of IgM on the cell membrane is significantly higher than that of IgG in B cells. However, as shown in Figure 6A, the expression level of IgG (mean ± SD, 28.6% ± 30.1%) was significantly higher than that of IgM (mean ± SD, 4.2% ± 4.9%) by flow cytometry, which was different from that of B cells. Next, V_H_DJ_H_ rearrangement patterns from different Ig classes were analyzed. For example, Igμ, Igα, and Igε in AML-Ig from AML3 tended to use IGHV4-59/IGHD3-16/IGHJ6 (141/1018, 13.8%), IGHV4-31/IGHD3-10/IGHJ3 (623/2680, 23.2%), and IGHV3-23/IGHD3-16/IGHJ6 (137/1058, 13.0%), respectively (Figure 6B, left). In addition, Igγ, Igμ, and Igα in AML-Ig from AML8 tended to use IGHV2-5/IGHD3-22/IGHJ3 (437/3709, 11.8%), IGHV3-74/IGHD2-15/IGHJ34 (219/523, 41.9%), and IGHV4-39/IGHD3-10/IGHJ4 (1197/4365, 27.4%), respectively (Figure 6B, right). Moreover, the same phenomenon was observed in AML-Ig from five other AML patients, where multiple Ig classes were detected (Figure 6C). Therefore, different Ig classes from the same patient tended to use different V_H_DJ_H_ rearrangement patterns, which may indicate that class-switch recombination may not exist in myeloblasts and that each Ig class has unique functions in the development and progression of AML.

### 3.6. Myeloid Cells at Different Differentiation Stages Express Different Ig Rearrangement Patterns

**Figure 7:**
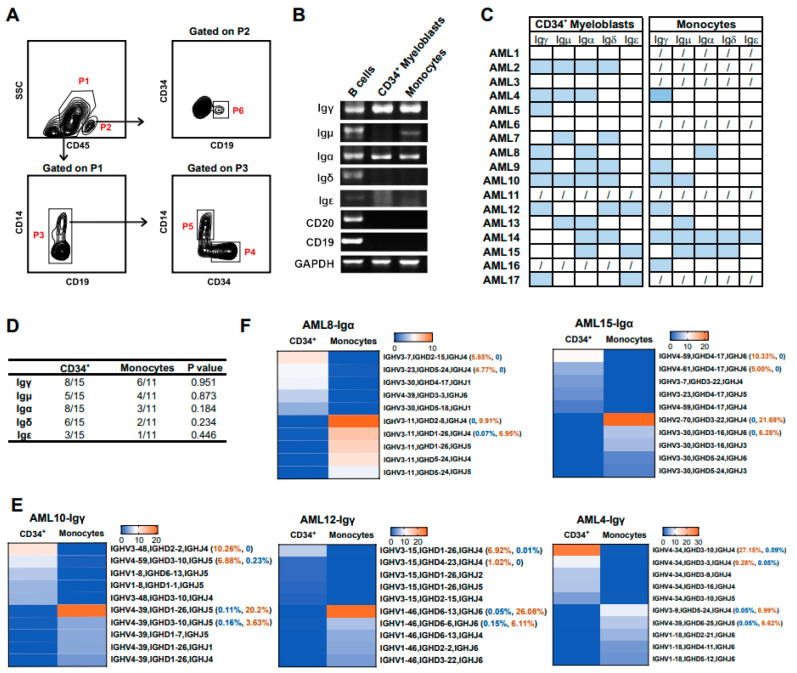
Myeloid cells at different differentiation stages expressed different Ig rearrangement patterns. (**A**) The representative gating strategy for myeloid cells. Myeloblasts (P4, CD45^+^/CD19^−^/CD14^−^/CD34^+^) and mature monocytes (P5, CD45^+^/CD19^−^/CD34^−^/CD14^+^) were obtained by gating on P3. B cells (P6, CD45^+^/CD19^+^) were obtained by gating on P2. (**B**) Representative PCR detection of Ig in CD34^+^ myeloblasts and mature monocytes. (**C,D**) Statistical analysis of Ig expression in CD34^+^ myeloblasts and mature monocytes from 17 AML patients. (**C**) The blue box represents Ig expression, the white box represents no Ig expression, and the forward slash indicates that the samples were not assessed. (**D**) Quantification of Ig expression in CD34^+^ myeloblasts and mature monocytes. The association between cell types and the expression of Ig was tested using the chi-square test. (**E**,**F**) The biased V(D)J rearrangement patterns were used by CD34^+^ myeloblasts and mature monocytes. Heatmaps showed the top five V(D)J rearrangement patterns of two classes of heavy chains, including γ (**E**) and α chains (**F**).

In order to further analyze the difference in Ig expression between myeloid cells at different stages of differentiation, myeloid cells were divided into two groups, including CD34^+^/CD14^−^ AML progenitor cells (CD34^+^ myeloblasts, P4 gate) and relatively mature CD34^−^/CD14^+^ cells (monocytes) (Figure 7A, P5 gate). B cells (CD19^+^CD34^−^ cells) were used as a positive control. The expression profile of Ig transcripts in CD34^+^ myeloblasts and relatively mature monocytes was shown by 5′-RACE, and both CD34^+^ myeloblasts and monocytes expressed five classes of IgH (Figure 7B,C). In addition, there was no difference between the expression frequency of IgH in CD34^+^ myeloblasts and monocytes (Figure 7D).

Through PacBio sequencing, a large number of Ig sequences from CD34^+^ myeloblasts and relatively mature monocytes were obtained, including Igγ (n = 71,257), Igμ (n = 3364), Igα (n = 38,894), Igδ (n = 10,300), and Igε (n = 4886) (Table 3). For Igγ and Igɑ, the biased usage of V(D)J rearrangement patterns was distinct between CD34^+^ myeloblasts and monocytes from the same patient (Figure 7E,F). For mature monocytes, the usages of the most prevalent VDJ rearrangements were usually more than 20%, except for Igγ in patient 17. However, for AML progenitor cells, the usages of top VDJ rearrangement were lower than 10%, except that of Igα in patient 15. Since the Igμ, Igδ, and Igε sequences were only detected in four cases, three cases, and one case, respectively, their sequence characteristics were not analyzed. These results indicated that myeloid cells at a more mature differentiation stage were more prone to clone proliferation than those at an immature stage.

### 3.7. The Biased Usage of V(D)J Rearrangement Patterns Are Different in the Same Ig Class of Myeloblasts from Different Individuals

**Figure 8:**
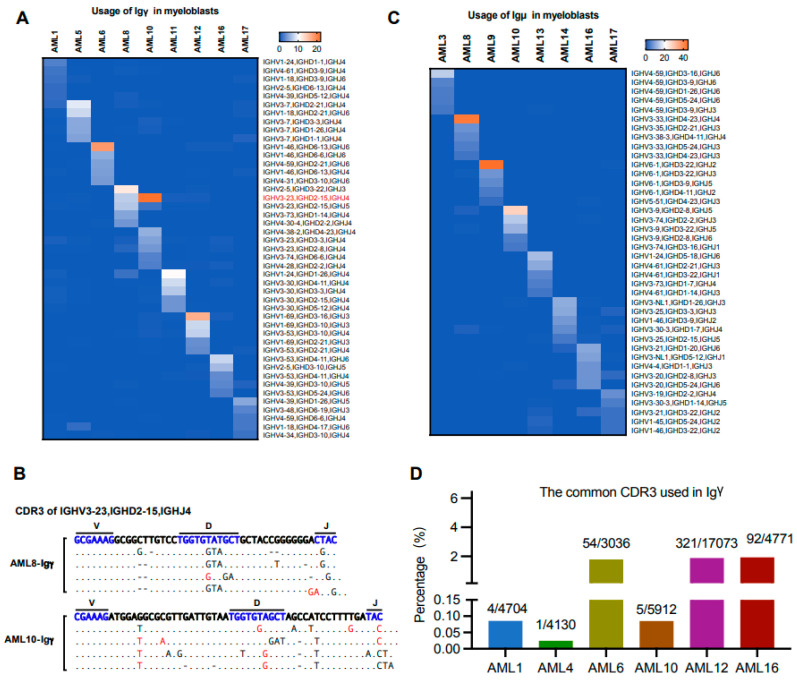
Characteristic Ig rearrangement patterns in the same Ig class expressed in different AML patients. (**A**) Heat maps showing the usage of the top five V(D)J rearrangement patterns in Igγ expressed by myeloblasts from different patients. The sequences marked in red are the common biased usage of V_H_DJ_H_ rearrangement patterns. (**B**) The CDR3 sequences of IGHV3-23/IGHD2-15/IGHJ4 from Igγ of AML8 and AML10 mutations (red, insertions; black, substitutions; hyphen, deletion). (**C**) Heat maps showing the usage of the top five V(D)J rearrangement patterns in Igμ expressed by myeloblasts from different patients. (**D**) The percentages of the special Igγ CDR3 used in 6 AML patients.

Previous studies have confirmed that IgG is highly conserved in solid tumors, and the same sequence was used among different patients, which could promote tumor cell proliferation and metastasis [10]. Till now, it is unclear whether the usage of V(D)J rearrangement patterns is biased among AML patients. As shown in Figure 8A, biased usage of V_H_DJ_H_ rearrangement patterns in AML-Igγ were different among nine patients, except that AML-Igγ from AML8 and AML10 shared IGHV3-23/IGHD2-15/IGHJ4. However, the CDR3 region sequences of IGHV3-23/IGHD2-15/IGHJ4 from AML8 and AML10 were different (Figure 8B). Similar results in Igγ were found in Figure 8C. Although the VDJ rearrangement patterns were different, a special CDR3 sequence (5′-gcgagagatcgagcagcagtccatacaactaccactacgctatggacgtc-3′) was found in 6/17 (35.3%) of AML patients (Figure 8D), indicating that this sequence may play a role in AML pathogenesis and progression.

## 4. Discussion

In this study, we performed a comparative analysis of the Ig repertoire between myeloblasts and B cells with the same individuals in a group of Chinese AML patients. We found that myelolasts could express all classes and types of Ig, with a particularly high frequency of IgG. Compared with B-Ig in the same patient, the biased V(D)J usage patterns of AML-Ig were different. In addition, the CDR3 length distribution of AML-Ig was significantly different from that of B-Ig. More importantly, the mutations of AML-IgH, especially Igμ, Igα, and Igδ, were different from B-IgH in each individual AML patient, which frequently occurred at the sites of post-translational modification.

Compared to our previous study [26], we selected newly diagnosed Chinese AML patients who had not received any treatment. In addition, the 5’-RACE was innovatively used in this study to obtain the Ig repertoire to avoid any bias caused by primers. Because the Ig variable region has high diversity, traditional Ig/BCR detection uses degenerate primers targeting the V region. The amplification efficiency of degenerate primers is different for distinct variable region sequences, which may lead to a biased tendency to amplify certain Ig variable regions. The 5′-RACE provides a mechanism for adding special nucleotide sequences to the 5′ end of the cDNA to amplify the accurate V sequences.

In keeping with our previous results [26], we found that myeloblasts expressed all five classes of IgH transcripts in AML, in particular with a high frequency of IgG expression. Moreover, the expression frequency of the five classes of IgH showed no significant difference between myeloblasts and B cells. However, the VDJ rearrangement patterns of AML-Ig were different from those of B-Ig within the same patient. All five classes of IgH exhibited biased V(D)J usage patterns, in contrast to the diversity of B-Ig. Moreover, there was a significant difference in the length of CDR3 between AML-Ig and B-Ig within the same patient, further indicating the disparity of the Ig repertoire between these two cell types. We have previously shown that the cancer-derived Ig gene repertoire showed specific restricted patterns of VDJ recombination with seven sets of predominant VDJ sequences in human epithelial cancer [45]. However, whereas biased VDJ rearrangements in epithelial cancers were found among different cancer patients, individual specificity of VDJ rearrangements was found in each AML patient.

Although the sample size is relatively small, the findings of the common CDR3 shared among AML patients suggest that the unique CDR3 sequence of AML-Ig may serve as a novel molecular marker for MRD monitoring and a therapeutic target. Indeed, our group has previously discovered that IgG can also be expressed in lung cancer cells and has a special sialylation modification (SIA-IgG). SIA-IgG reciprocally stimulated SOX2 by activating the c-Met/Akt/Erk signaling axis, constituting a self-propagating loop of SIA-IgG/c-Met/SOX2/SIA-IgG signaling, which is crucial for cancer stemness. We subsequently developed a monoclonal antibody, RP215, which specifically recognizes the Asn162 sialylation-related epitope on SIA-IgG, effectively blocked the SIA-IgG-driven signaling loop, and inhibited lung cancer cell stemness and tumor growth [2]. A study is underway using a similar approach to develop a monoclonal antibody or small molecular inhibitor for the treatment of AML.

The sequence characteristic of Ig in the myeloid cells at different stages of differentiation in each patient was also different. The usage of most of the prevalent VDJ rearrangements was usually greater than 20% in mature monocytes but less than 10% in myeloblasts. These results may suggest that myeloid cells with the Ig gene at a more mature differentiation stage were more prone to diversity than cells in an immature stage.

In this study, we also found that AML-Ig showed unique sites of somatic hypermutation (SHM) compared with B-Ig. It is known that B cell-derived Igμ and Igδ heavy chain variable regions do not undergo SHM, while Igγ, Igα, and Igε heavy chain variable regions have a higher frequency of mutations [46,47]. However, SHM occurred in all classes and types of AML-Ig variable regions. When AML-Ig and B-Ig used the same V segment, AML-Ig had unique SHM sites, and these sites tended to occur at specific amino acids, which might affect post-translational modifications of Ig. In particular, the mutations in S and T might affect O-glycosylation modifications in AML-IgH. This discovery suggests that these unique mutation patterns may have significant implications for the pathogenesis of AML and require further exploration.

In addition, the AML-Ig showed unique sites of SHM that may also be used for monitoring MRD. In our previous study, we focused on Ig light chains and found special S mutation hotspots in AML-derived IGKV3-20 among AML patients, which differed from classical IGK in B cells [1]. The conserved mutation may serve as a molecular marker for MRD monitoring and the development of target therapies for AML. In this study, we focused on Ig heavy chains and identified several S mutations with significant differences between AML-Ig and B-Ig in the same patient, even in healthy individuals. We also analyzed the mutation frequencies and patterns of Ig among AML patients and found that each individual exhibited unique mutation characteristics in AML-derived Ig, which may become indicators of individualized MDR.

## 5. Conclusions

In summary, we have found that myelolasts could express all classes and types of Ig, with a particularly high frequency of IgG. Compared with B-Ig in the same AML patient, the biased V(D)J usage and mutation patterns of AML-Ig were different. More importantly, the mutation of AML-Ig frequently occurred at the sites of post-translational modification. The unique V(D)J usage of AML-Ig may serve as a novel biomarker for personalized MRD monitoring and therapeutic targets in AML patients.

## Figures and Tables

**Table 1:** Clinicopathologic, cytogenetic, and molecular features of 27 patients with acute myeloid leukemia.

Patient ID	Sex	Age (year)	2022 WHO Classification	BM Blasts (%)	*FLT3*-ITD Mutation	*NPM1* Mutation	EVI1 Overexpression	*MLL*-PTD	*WT1* Mutation
AML1	F	40	AML with *NPM1* mutation	49	+	+	-	-	-
AML2	F	35	AML with maturation	55	-	-	-	-	-
AML3	F	58	AML with maturation	52	-	-	-	-	-
AML4	F	45	AML with *RUNX1::RUNX1T1*	33	-	-	-	-	+
AML5	F	51	AML with maturation	79	-	-	+	+	-
AML6	M	23	AML with *RUNX1::RUNX1T1*	25	-	-	-	-	-
AML7	M	61	AML with maturation	53	-	-	-	+	+
AML8	M	51	AML with maturation	72	-	-	-	-	+
AML9	M	37	AML with maturation	86	-	-	-	-	+
AML10	M	31	AML with maturation	63	+	-	-	-	+
AML11	M	61	AML with maturation	41	-	-	-	-	+
AML12	F	65	AML with maturation	57	-	-	-	+	+
AML13	M	31	AML with maturation	58	-	-	-	-	+
AML14	M	18	Acute monocytic leukemia	71	+	-	-	+	+
AML15	F	31	AML with *MECOM* rearrangement	59	-	-	+	-	+
AML16	F	38	AML with *NPM1* mutation	53	-	+	-	-	+
AML17	F	62	Acute myelomonocytic leukemia	85	-	-	-	-	-
AML18	F	60	Acute myelomonocytic leukemia	44	-	-	-	-	-
AML19	M	36	AML with maturation	41	-	-	+	+	-
AML20	M	67	AML with maturation	NA	-	-	+	+	-
AML21	F	54	AML with *KMT2A* rearrangement	NA	-	-	-	-	-
AML22	M	83	AML with maturation	86	+	+	-	-	-
AML23	F	60	AML with maturation	54	-	+	-	-	+
AML24	M	52	AML with *NPM1* mutation	41	-	+	-	-	+
AML25	F	50	APL with *PML::RARA*	66	-	-	-	-	+
AML26	M	54	AML with maturation	27	-	-	-	+	+
AML27	F	35	APL with *PML::RARA*	79	-	-	-	-	-

AML, acute myeloid leukemia; BM, bone marrow; F, female; M, male; NA, not available; +, present; -, absent.

**Table 2:** The counts of Ig sequences in myeloblasts and B cells from 17 AML patients.

Patient ID	Sorted Cells	Igγ	Igμ	Igα	Igδ	Igε
AML1	Myeloblasts	4704	NA	NA	NA	NA
	B cells	1356	2030	1148	2833	NA
AML2	B cells	1725	911	NA	2316	NA
AML3	Myeloblasts	NA	1018	2680	NA	1058
	B cells	3991	2416	3113	1481	NA
AML4	B cells	814	1401	317	1155	NA
AML5	Myeloblasts	12,377	NA	NA	NA	NA
	B cells	1877	NA	893	NA	NA
AML6	Myeloblasts	3036	NA	NA	NA	NA
	B cells	2820	1343	4236	4545	244
AML7	Myeloblasts	NA	NA	NA	NA	NA
	B cells	4425	1226	3202	NA	NA
AML8	Myeloblasts	3709	523	4365	NA	NA
	B cells	9586	NA	NA	NA	NA
AML9	Myeloblasts	NA	5583	5848	NA	NA
	B cells	457	1027	1737	3463	NA
AML10	Myeloblasts	4853	1355	2625	NA	NA
	B cells	2179	1127	3120	2095	NA
AML11	Myeloblasts	3724	NA	NA	NA	NA
AML12	Myeloblasts	7250	NA	NA	NA	NA
	B cells	5628	2218	3104	NA	666
AML13	Myeloblasts	NA	880	1550	1651	NA
	B cells	NA	1576	894	2123	1499
AML14	Myeloblasts	NA	293	2155	295	NA
	B cells	NA	1402	NA	1685	NA
AML15	Myeloblasts	NA	NA	1085	NA	NA
	B cells	NA	871	477	1681	NA
AML16	Myeloblasts	1550	865	2981	2876	NA
	B cells	522	1921	925	2166	NA
AML17	Myeloblasts	1472	864	1374	3181	NA
	B cells	617	504	2822	854	5632
Total		78,672	31,354	50,651	34,400	9099

NA, not applicable.

**Table 3:** The counts of Ig sequences of CD34^+^ myeloblasts and monocytes from 14 AML patients.

Patient ID	Sorted Cells	Igγ	Igμ	Igα	Igδ	Igε
AML2	Myeloblasts	1752	NA	1775	NA	NA
AML4	Myeloblasts	3690	1640	1478	NA	N/A
	Monocytes	4130	NA	317	NA	NA
AML5	Myeloblasts	4643	NA	NA	NA	NA
AML8	Myeloblasts	4611	NA	2775	NA	NA
	Monocytes	NA	NA	6112	NA	NA
AML9	Myeloblasts	NA	NA	6493	6908	NA
	Monocytes	9215	NA	NA	NA	NA
AML10	Myeloblasts	3486	NA	4114	61	NA
	Monocytes	5912	NA	NA	NA	NA
AML12	Myeloblasts	7804	NA	NA	NA	NA
	Monocytes	17,073	NA	NA	NA	NA
AML13	Myeloblasts	NA	468	3198	NA	NA
	Monocytes	NA	266	1757	3266	NA
AML15	Myeloblasts	NA	NA	6865	NA	NA
	Monocytes	NA	990	4010	65	NA
AML16	Monocytes	4771	NA	NA	NA	NA
AML17	Myeloblasts	4170	NA	NA	NA	4886
Total		71,257	3364	38,894	10,300	4886

NA, not applicable.

## Data Availability

Immune repertoire data have been deposited in the Sequence Read Archive (SRA) under the accession code PRJNA725343.

## Supplementary Materials

The following supporting information can be downloaded at: https://www.mdpi.com/article/10.3390/biology13080613/s1, Figure S1: The CDR3 length distribution of IgH in myeloblasts and B cells. The CDR3 length distribution of IgH in Myeloblasts and B cells from 8 AML patients. Figure S2: The biased VHDJH rearrangement patterns of Igγ and Igμ in myeloblasts and B cells from AML patients. Figure S3:The biased VHDJH rearrangement patterns of Igα and Igδ in myeloblasts and B cells from AML patients. Figure S4. The distribution on the genome of the used VH gene in myeloblasts and B cells from 5 patients, including AML1, AML3, AML5, AML6 and AML8. Figure S5. The distribution on the genome of the used VH gene in myeloblasts and B cells from 5 patients, including AML9, AML10, AML12, AML13 and AML14. Figure S6. The distribution on the genome of the used VH gene in myeloblasts and B cells. Figure S7. Comparisons of VH mutation sites among myeloblasts, B cells from the same patient (AML-B cells), and B cell from healthy donors (Healthy donors-B cells). Table S1: The sequences of PCR primers used in this study.
