# Supplementary material for: A Comparative Analysis of the Immunoglobulin Repertoire in Leukemia Cells and B Cells in Chinese Acute Myeloid Leukemia by High-Throughput Sequencing
Source: Biology (Basel). 2024 Aug 13;13(8):613. doi: 10.3390/biology13080613 (PMC11351582; doi:10.3390/biology13080613)
Supplement: Supplementary file 1 [file biology-13-00613-s001.zip › Supplemental Materials_3109340.pdf]

Supplementary Figures

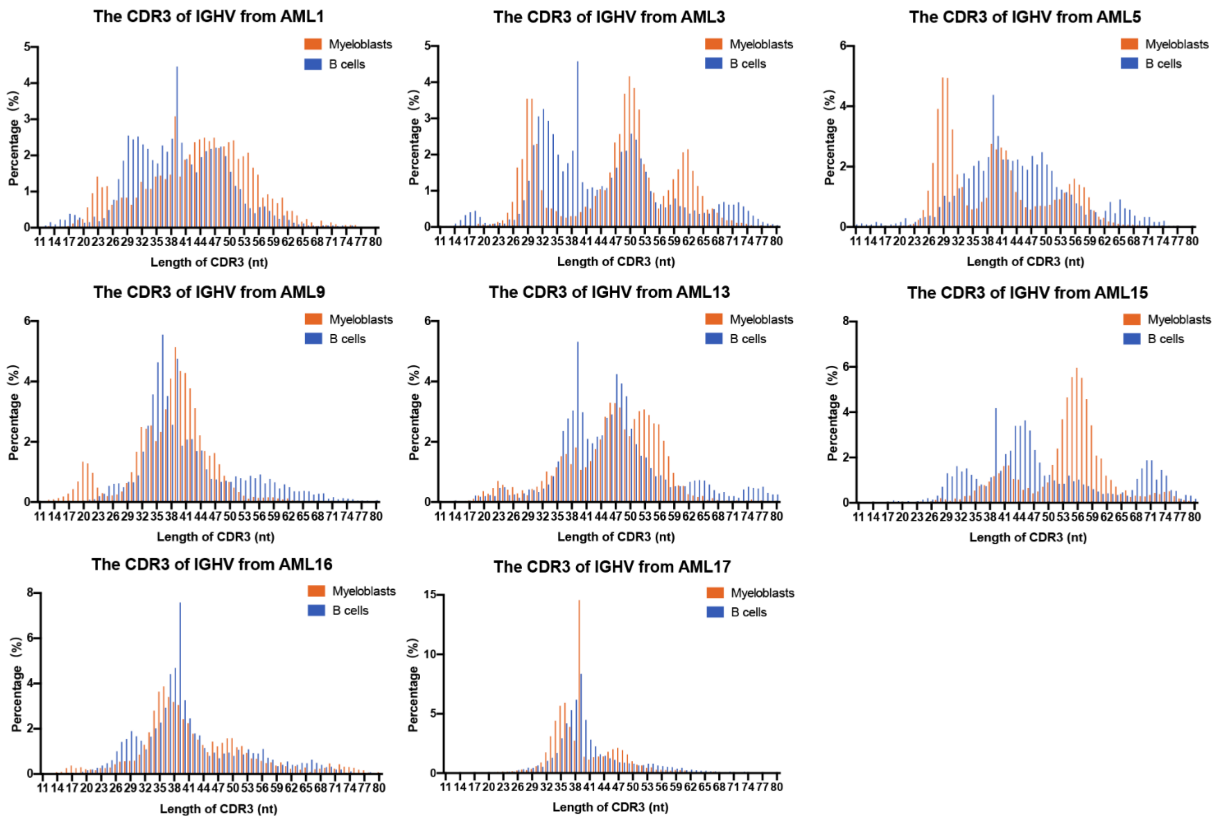

Figure S1. The CDR3 length distribution of IgH in myeloblasts and B cells. The CDR3 length distribution of IgH in Myeloblasts and B cells from 8 AML patients.

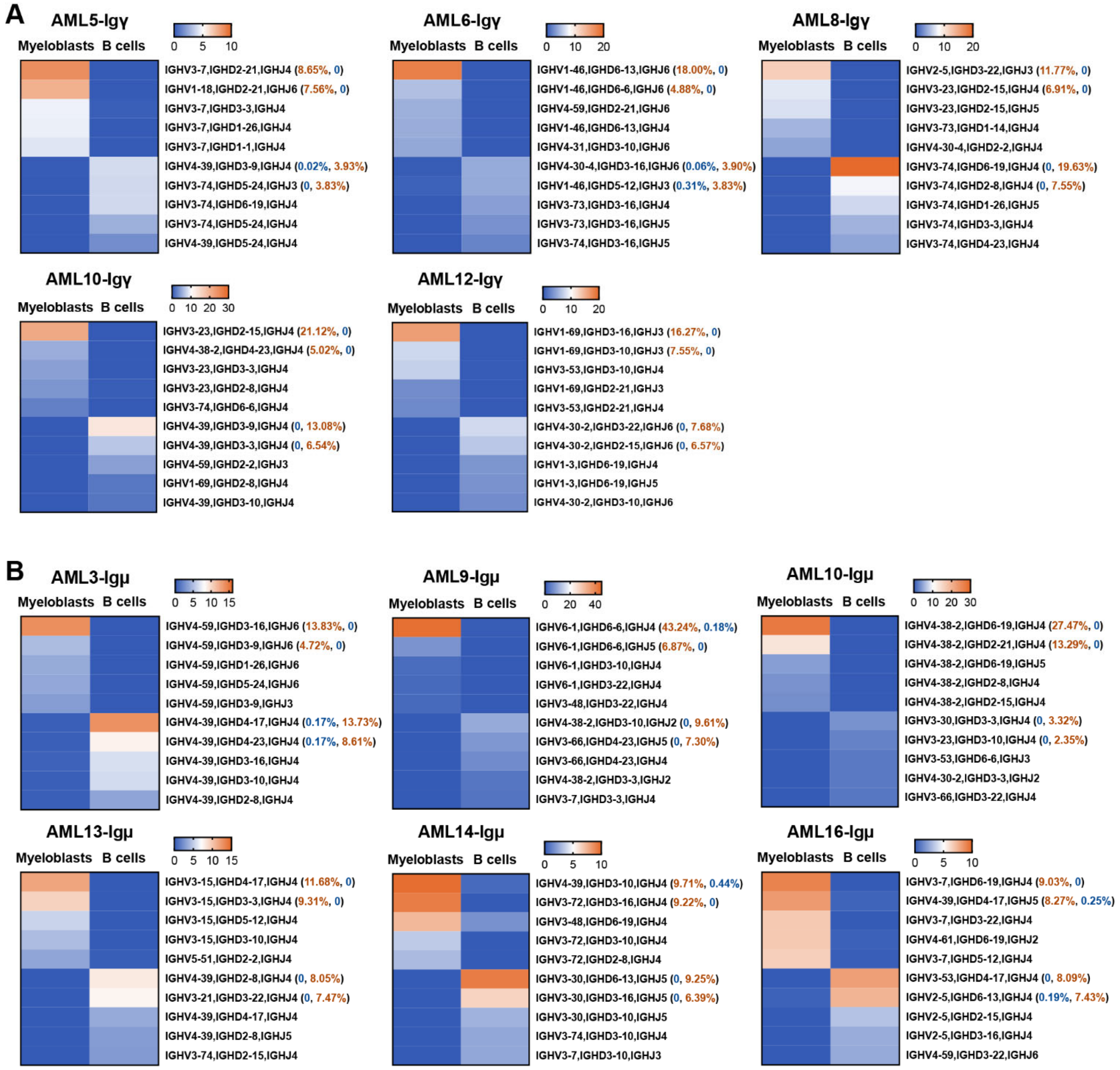

Figure S2. The biased V_HDJ_H rearrangement patterns of Igγ and Igμ in myeloblasts and B cells from AML patients. **A** The top 5 V_HDJ_H rearrangement patterns of Igγ in myeloblasts and B cells from 5 AML patients. **B** The top 5 V_HDJ_H rearrangement patterns of Igμ in myeloblasts and B cells from 6 AML patients.

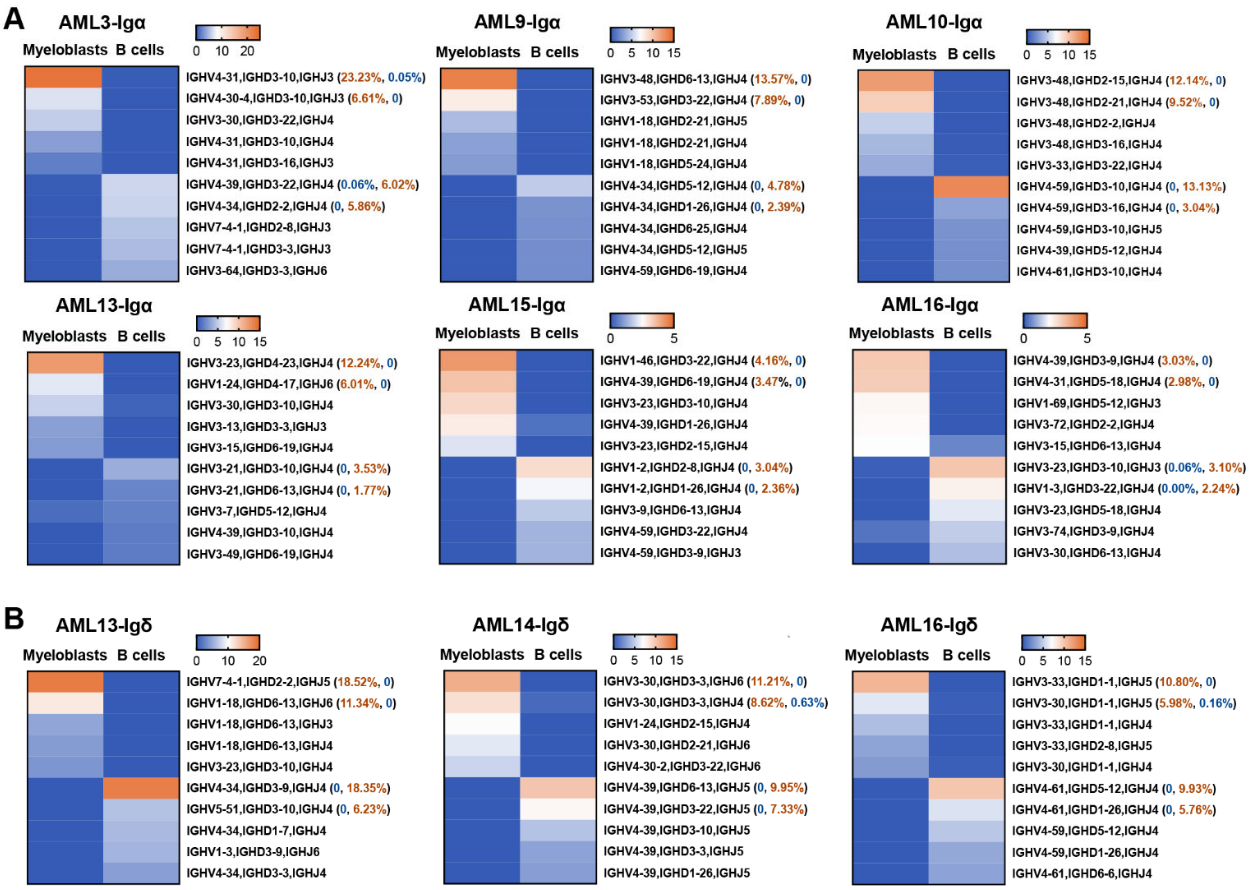

Figure S3. The biased V_HDJ_H rearrangement patterns of Igα and Igδ in myeloblasts and B cells from AML patients. **A** The top 5 V_HDJ_H rearrangement patterns of Igα in myeloblasts and B cells from 6 AML patients. **B** The top 5 V_HDJ_H rearrangement patterns of Igδ in myeloblasts and B cells from 3 AML patients.

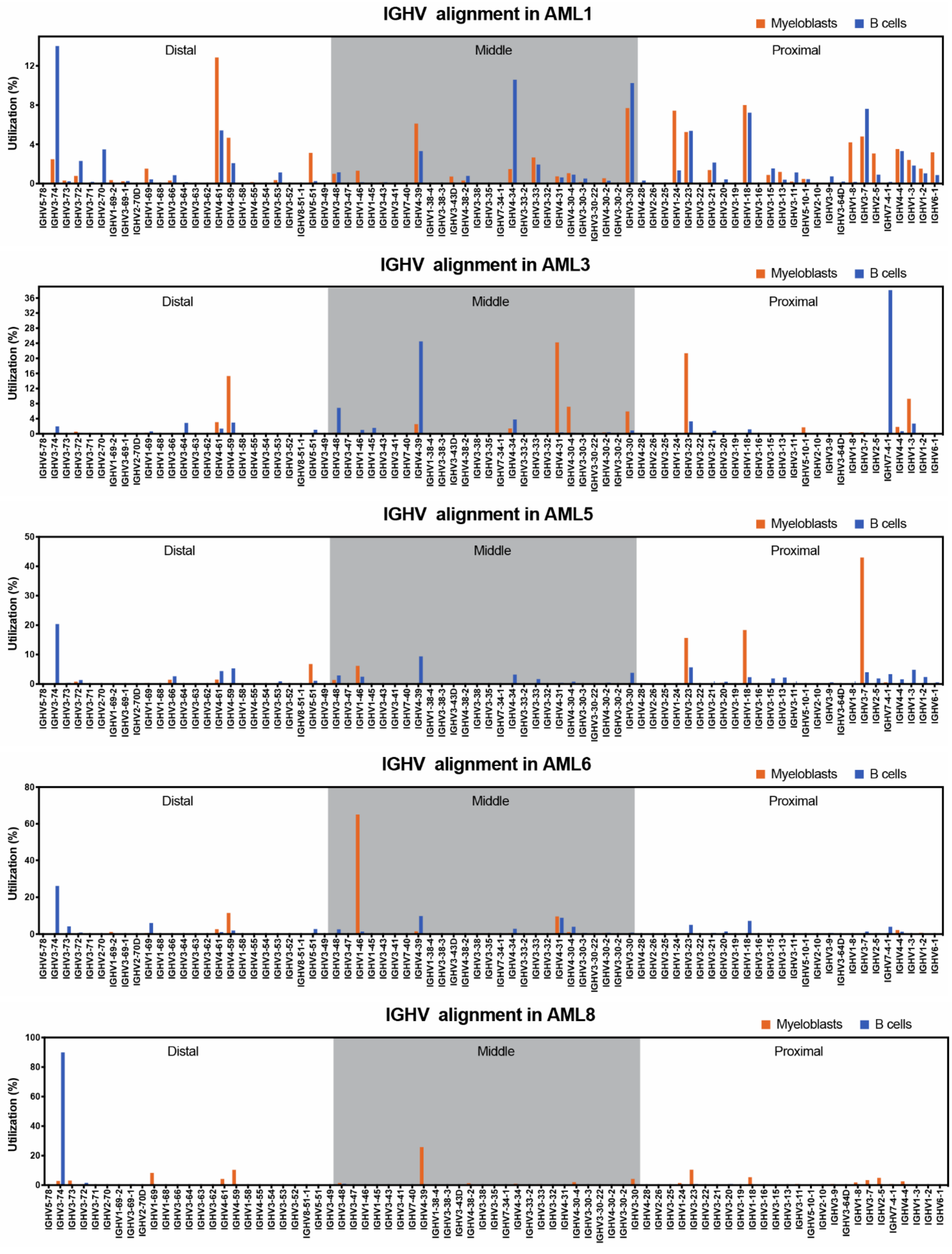

Figure S4. The distribution on the genome of the used V_H gene in myeloblasts and B cells from 5 patients, including AML1, AML3, AML5, AML6 and AML8. X-axis showed the order of V_H gene on the genome. Y-axis showed the percentage of used V_H gene in myeloblasts and B cells. Distal, middle and proximal represents the relative distance from J region and the V_H gene in proximal are closer to the J region on the genome.

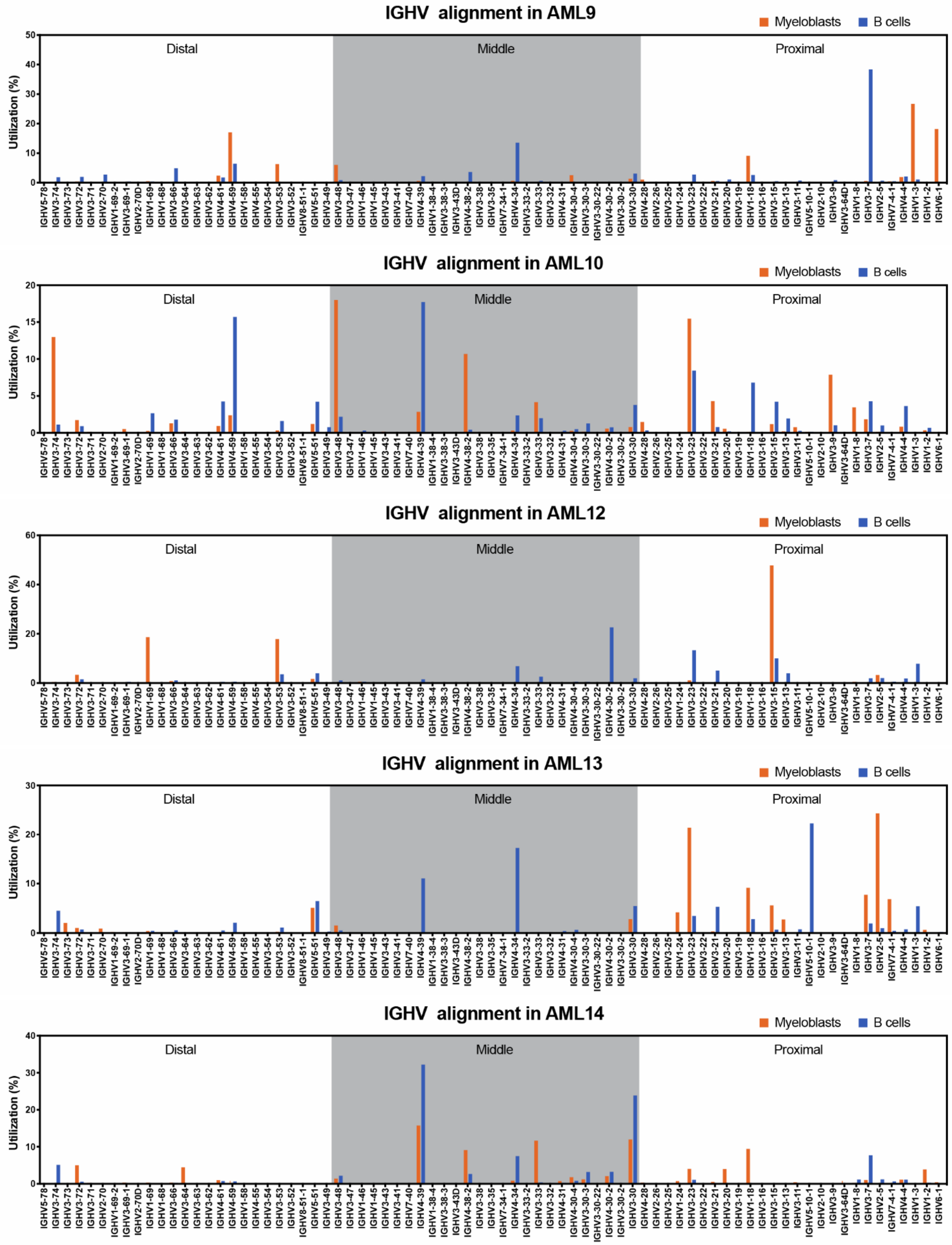

Figure S5. The distribution on the genome of the used V_H gene in myeloblasts and B cells from 5 patients, including AML9, AML10, AML12, AML13 and AML14.

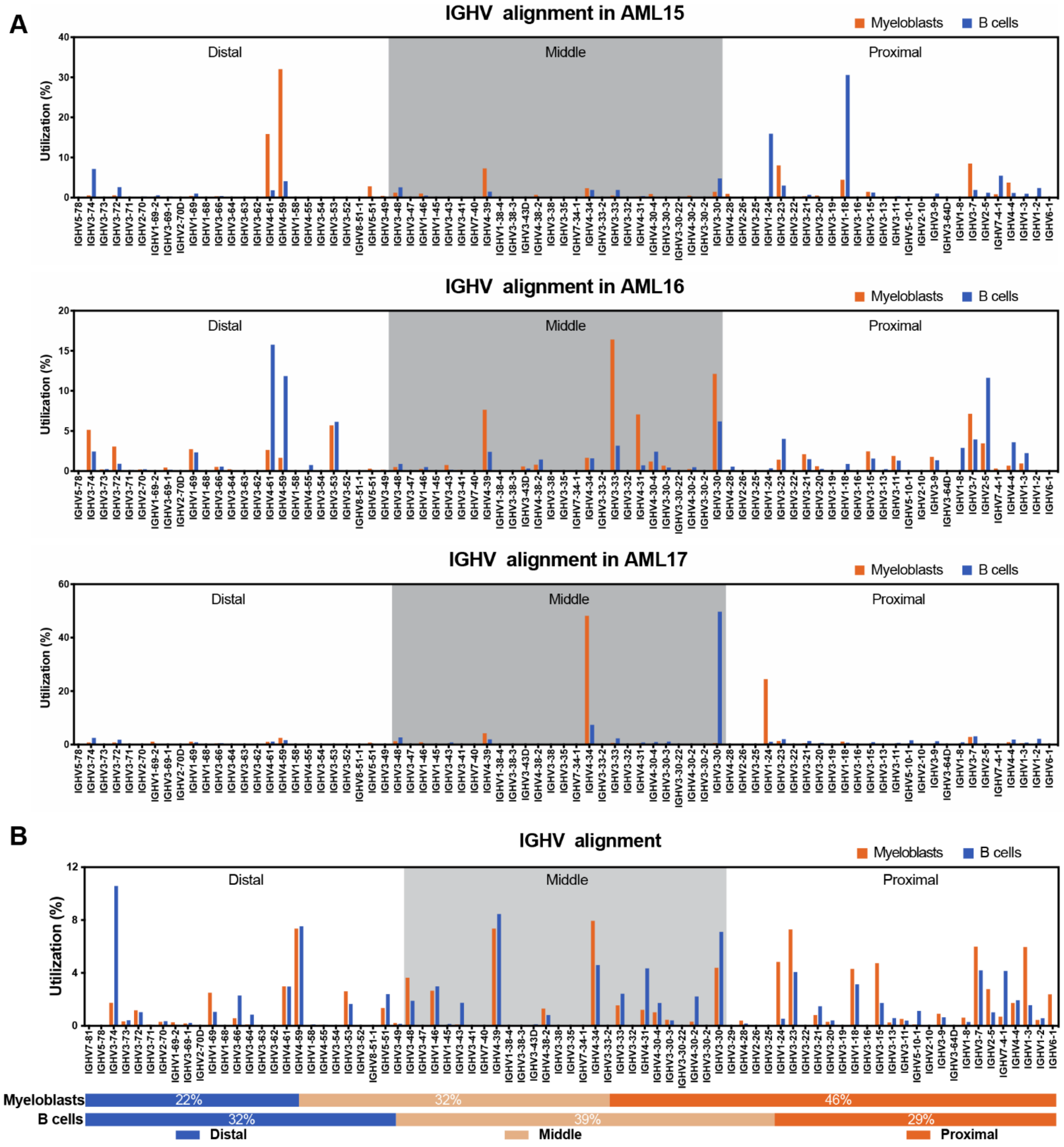

Figure S6. The distribution on the genome of the used V_H gene in myeloblasts and B cells. **A** The distribution on the genome of the used V_H gene in myeloblasts and B cells from 4 patients, including AML15-17. **B** The distribution on the genome of the all V_H gene in myeloblasts and B cells from 13 AML patients.

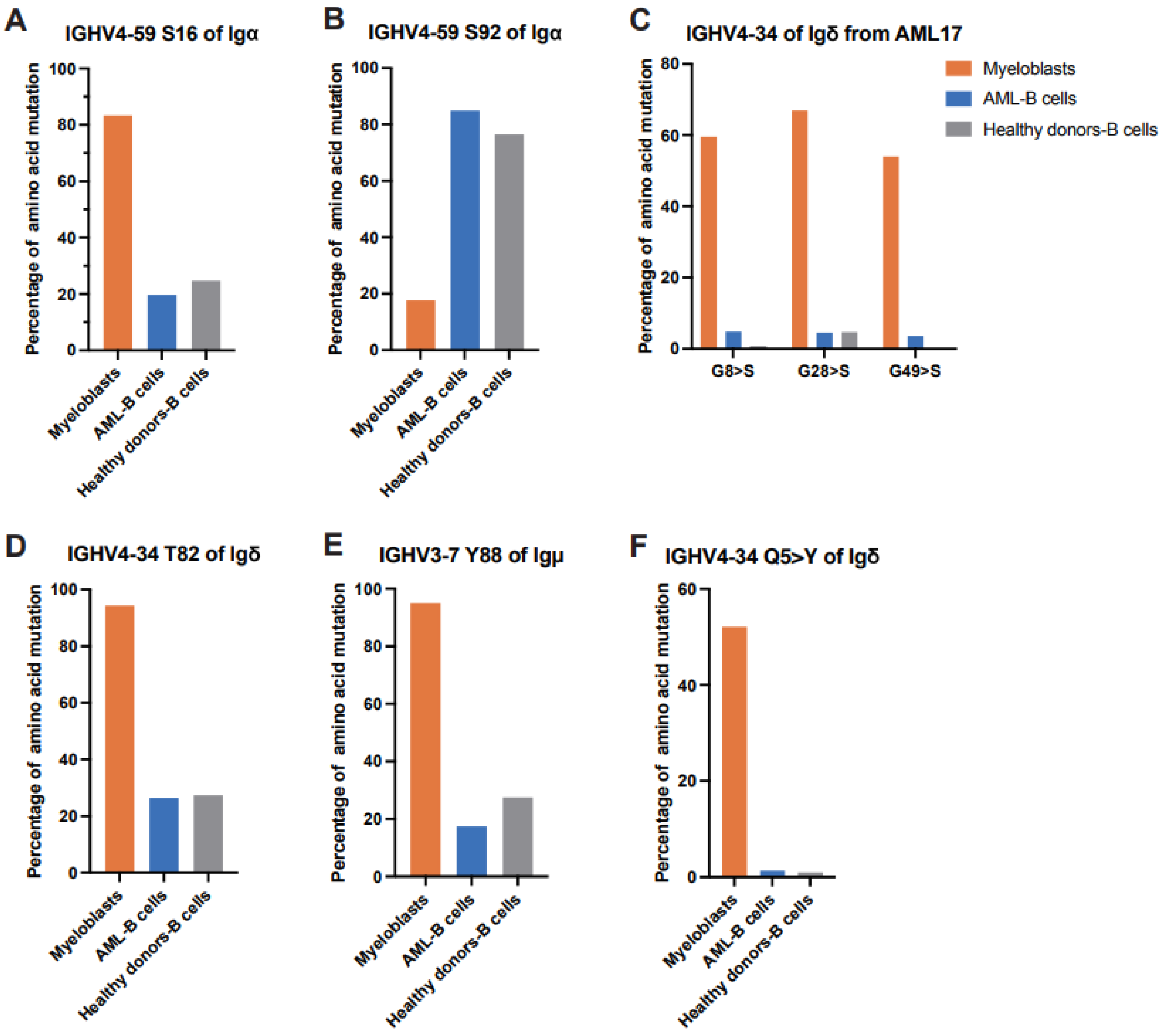

Figure S7. Comparisons of VH mutation sites among myeloblasts, B cells from the same patient (AML-B cells), and B cell from healthy donors (Healthy donors-B cells). (A) IGH4-59 S16 of Igα. (B) IGH4-59 S92 of Igα. (C) IGHV4-34 of Igδ. (D) IGH4-34 T82 of Igδ. (E) IGHV3-7 Y88 of Igμ. (F) IGHV4-34 Q5>Y of Igδ.
